# Supplementary figures and images for: Osteosarcoma-enriched transcripts paradoxically generate osteosarcoma-suppressing extracellular proteins
Source: eLife. 2023 Mar 21;12:e83768. doi: 10.7554/eLife.83768 (PMC10030111; doi:10.7554/eLife.83768)

## Slide 1
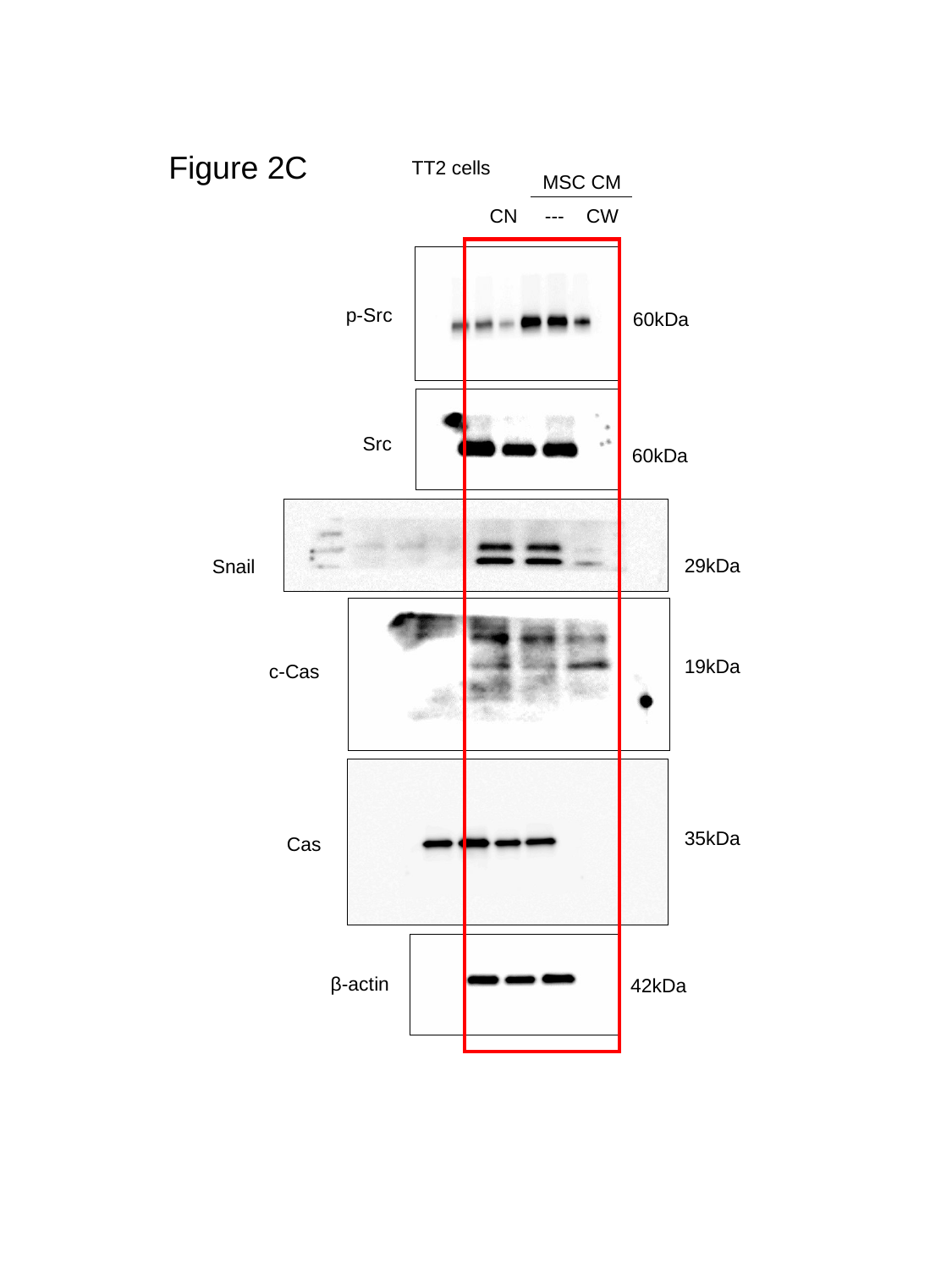

Figure 2C
TT2 cells
MSC CM
CN --- CW
p-Src
60kDa
Src
60kDa
29kDa
Snail
19kDa
c-Cas
35kDa
Cas
β-actin
42kDa

Supplement: Figure 2—source data 1. [file elife-83768-fig2-data1.zip › Figure 2-source data/Figure 2C-source data 1/Figure 2C-source data 7.pptx]

## Slide 1
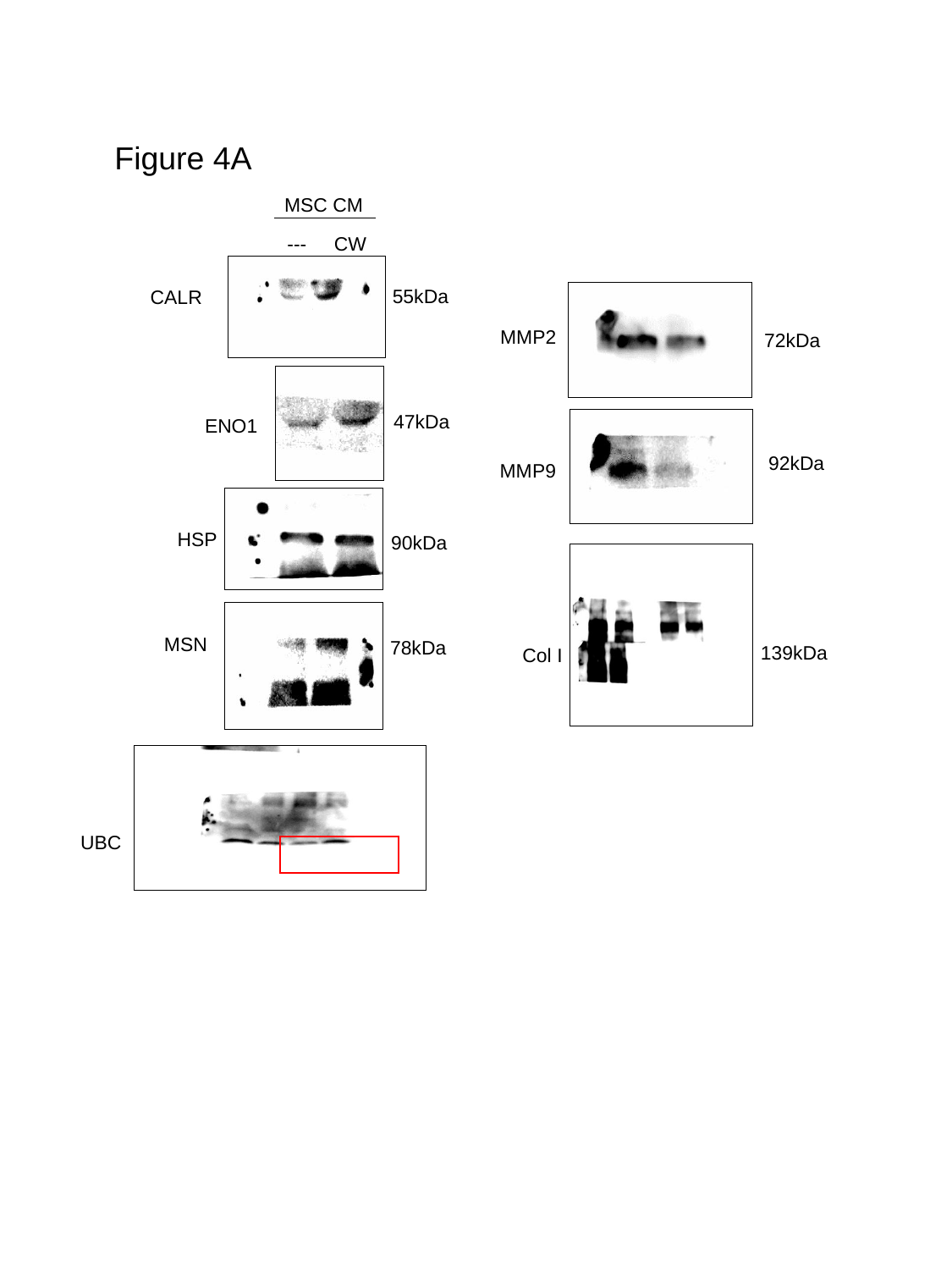

Figure 4A
MSC CM
--- CW
55kDa
CALR
MMP2
72kDa
47kDa
ENO1
92kDa
MMP9
HSP
90kDa
MSN
78kDa
139kDa
Col I
UBC

Supplement: Figure 4—source data 1. [file elife-83768-fig4-data1.zip › Figure 4-source data/Figure 4A-source data 1/Figure 4A-source data 9.pptx]

## Slide 1
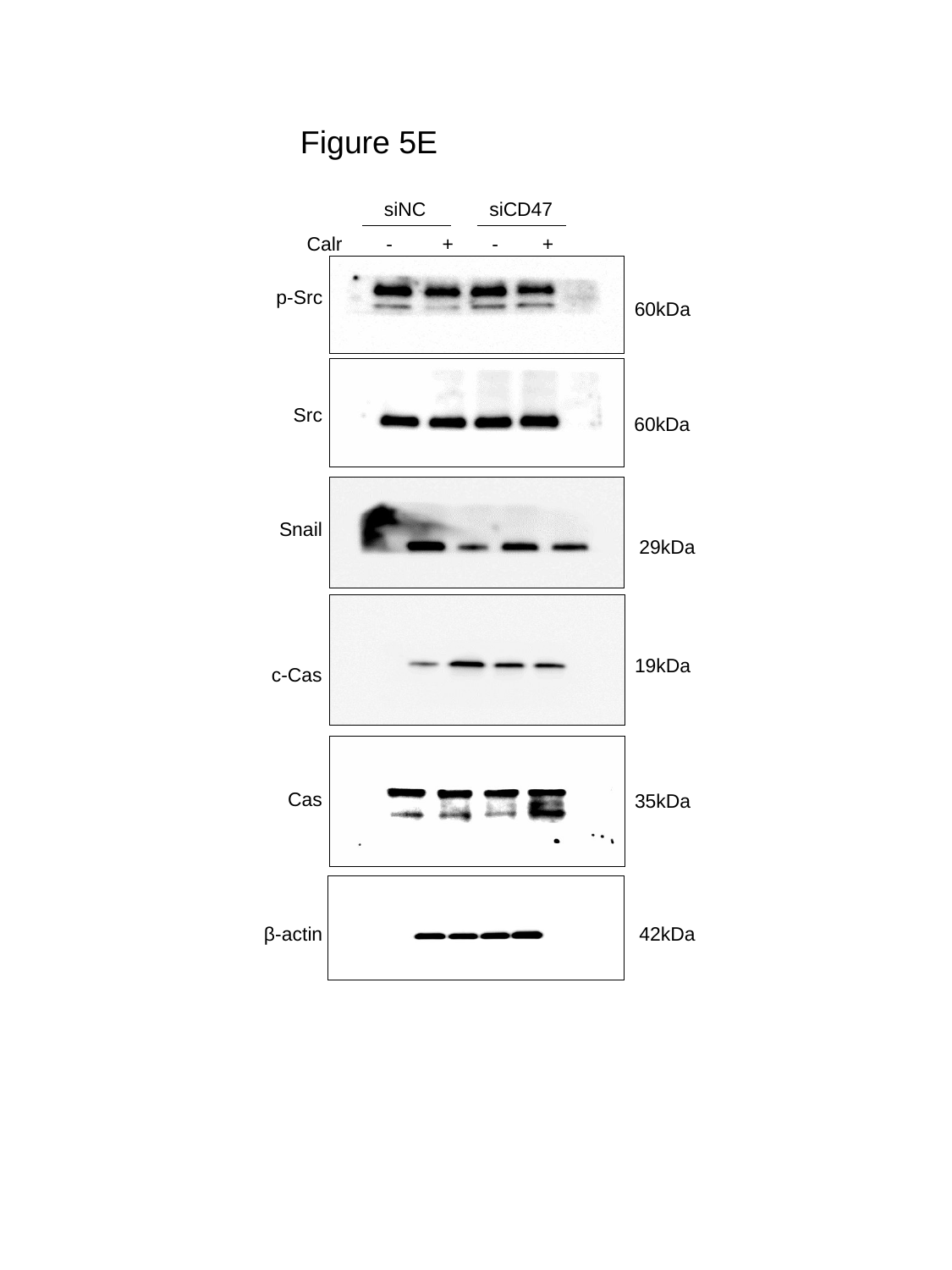

Figure 5E
siNC
siCD47
Calr - + - +
p-Src
60kDa
Src
60kDa
Snail
29kDa
19kDa
c-Cas
Cas
35kDa
42kDa
β-actin

Supplement: Figure 5—source data 1. [file elife-83768-fig5-data1.zip › Figure 5-source data/Figure 5E-source data 1/Figure 5E-source data 7.pptx]

## Slide 1
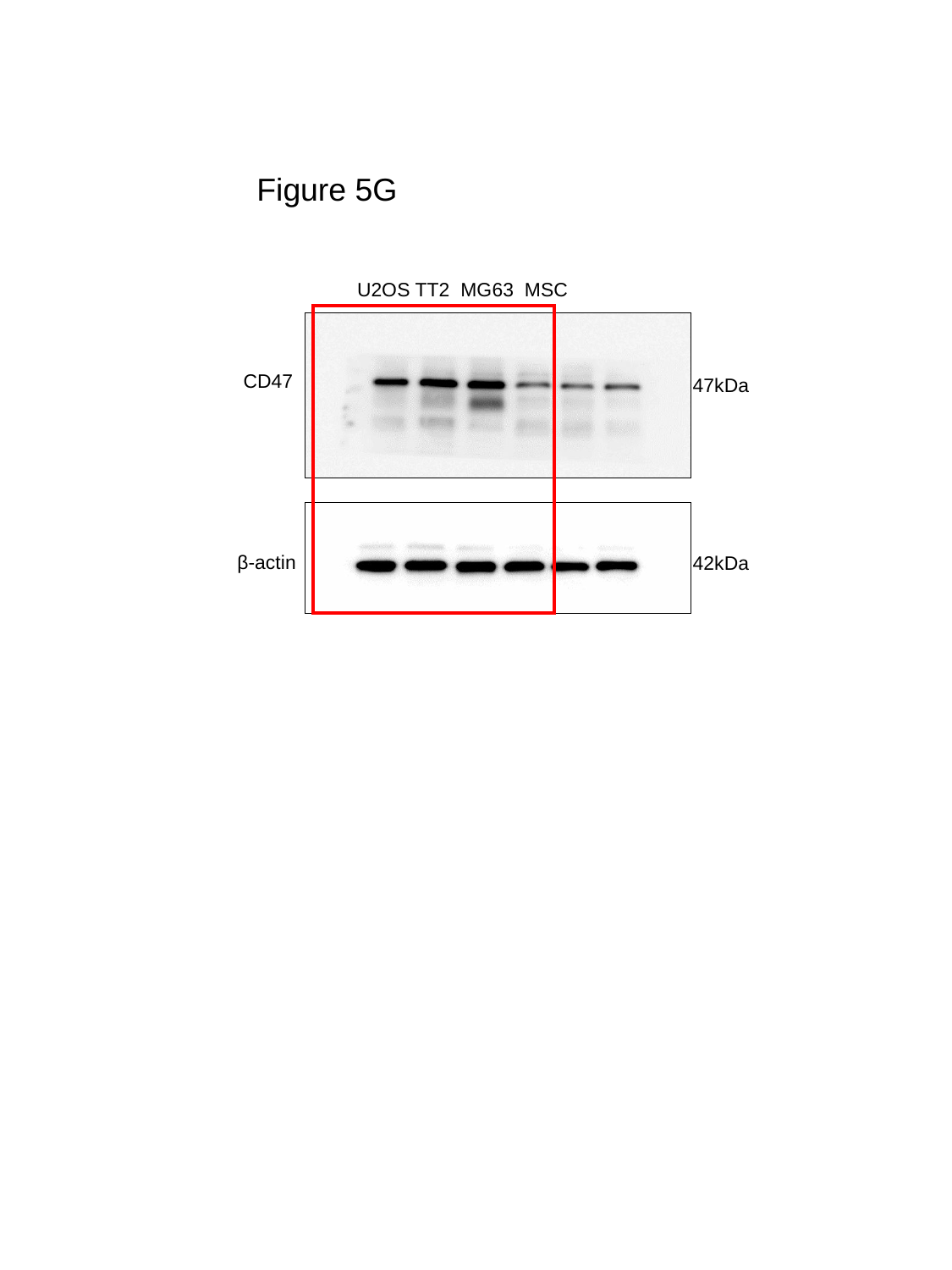

Figure 5G
U2OS TT2 MG63 MSC
CD47
47kDa
β-actin
42kDa

Supplement: Figure 5—source data 1. [file elife-83768-fig5-data1.zip › Figure 5-source data/Figure 5G-source data 1/Figure 5G-source data 3.pptx]

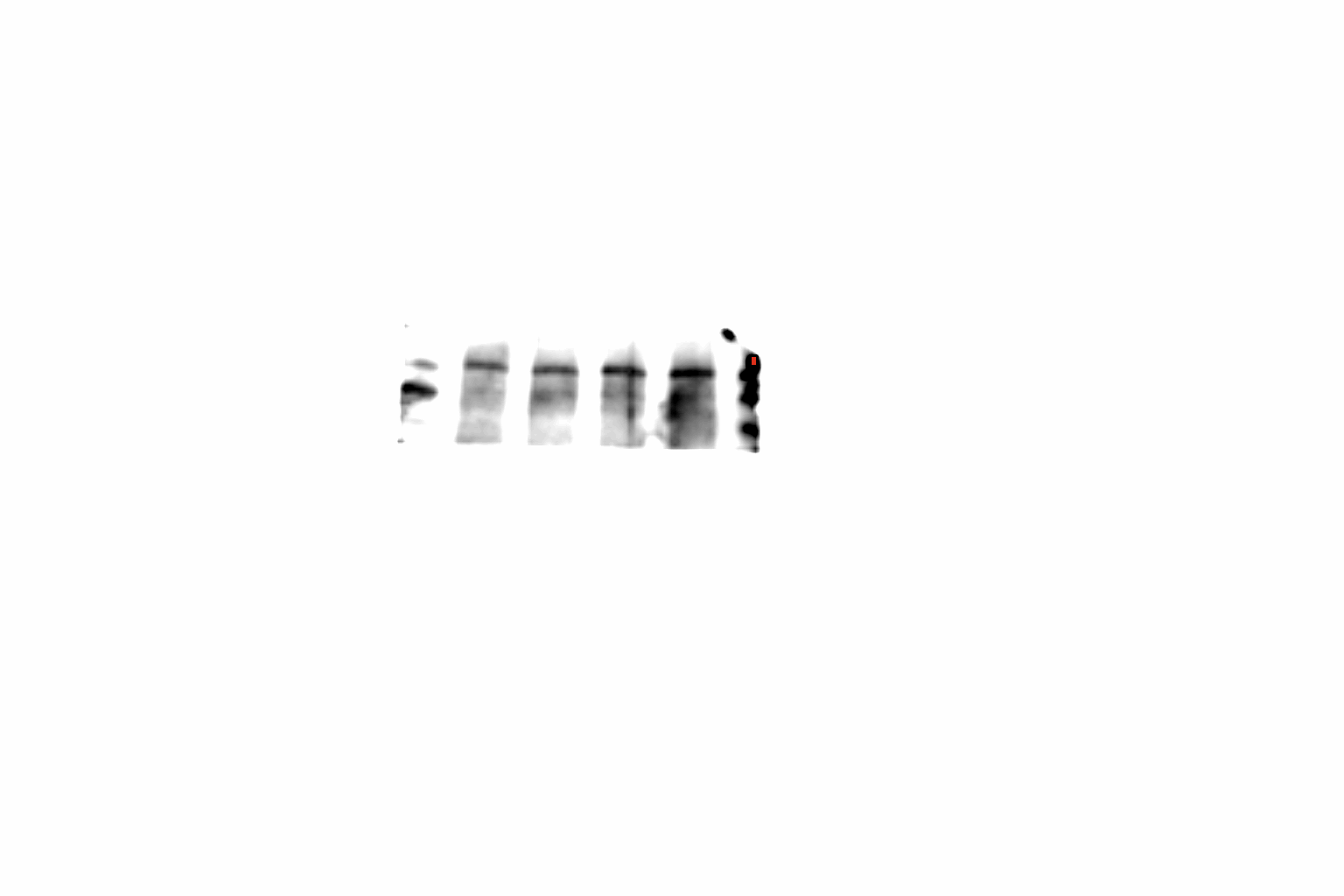

Supplement: Figure 5—figure supplement 2—source data 1. [file elife-83768-fig5-figsupp2-data1.zip › Figure 5-figure supplement 2-source data/Figure 5-figure supplement 2-source data 1/Figure 5-figure supplement 2-source data 1-COL I.tif]

## Slide 1
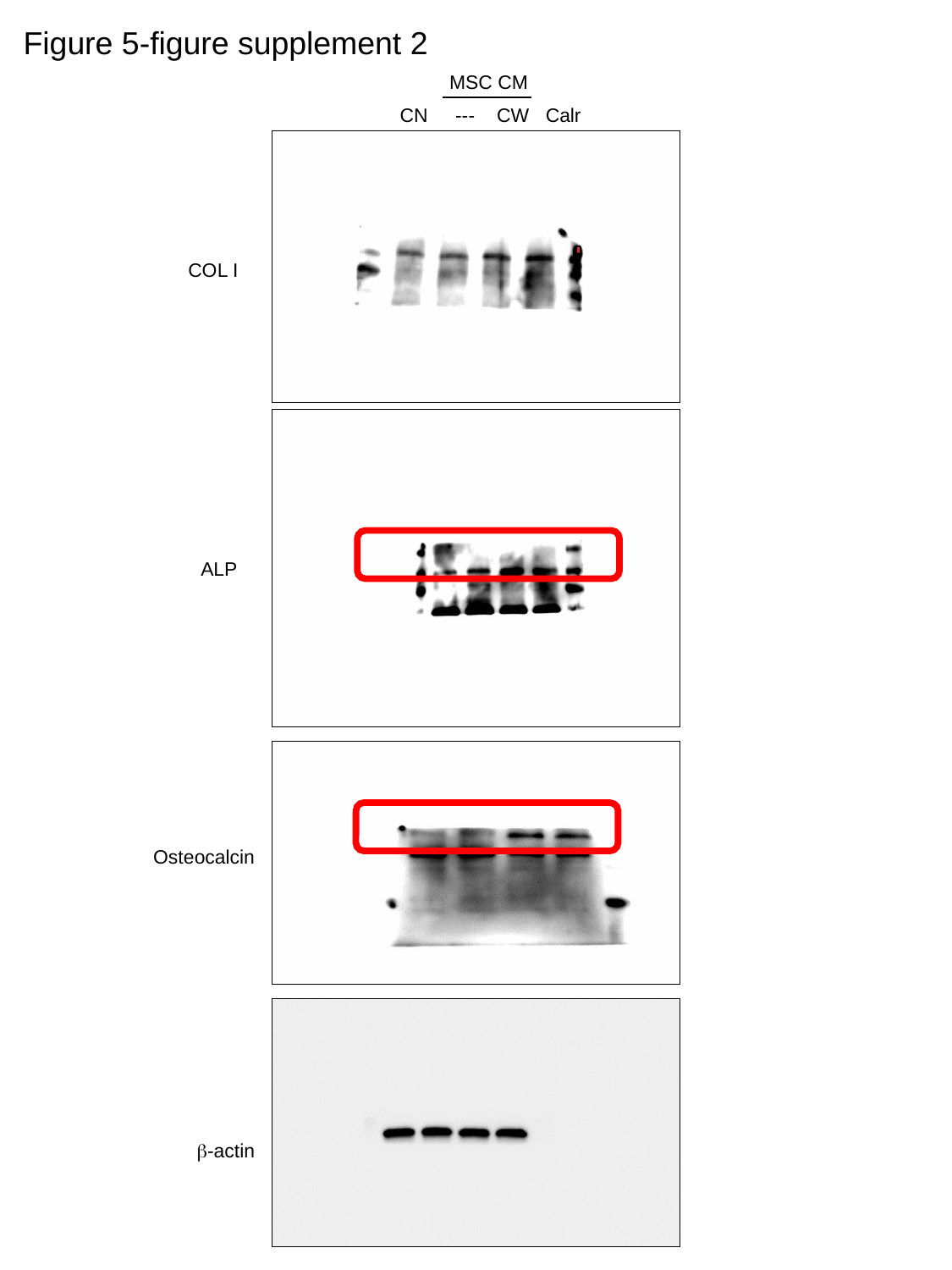

Figure 5-figure supplement 2
 MSC CM
CN --- CW Calr
COL I
ALP
Osteocalcin
b-actin

Supplement: Figure 5—figure supplement 2—source data 1. [file elife-83768-fig5-figsupp2-data1.zip › Figure 5-figure supplement 2-source data/Figure 5-figure supplement 2-source data 1/Figure 5-figure supplement 2-source data.pptx]

## Slide 1
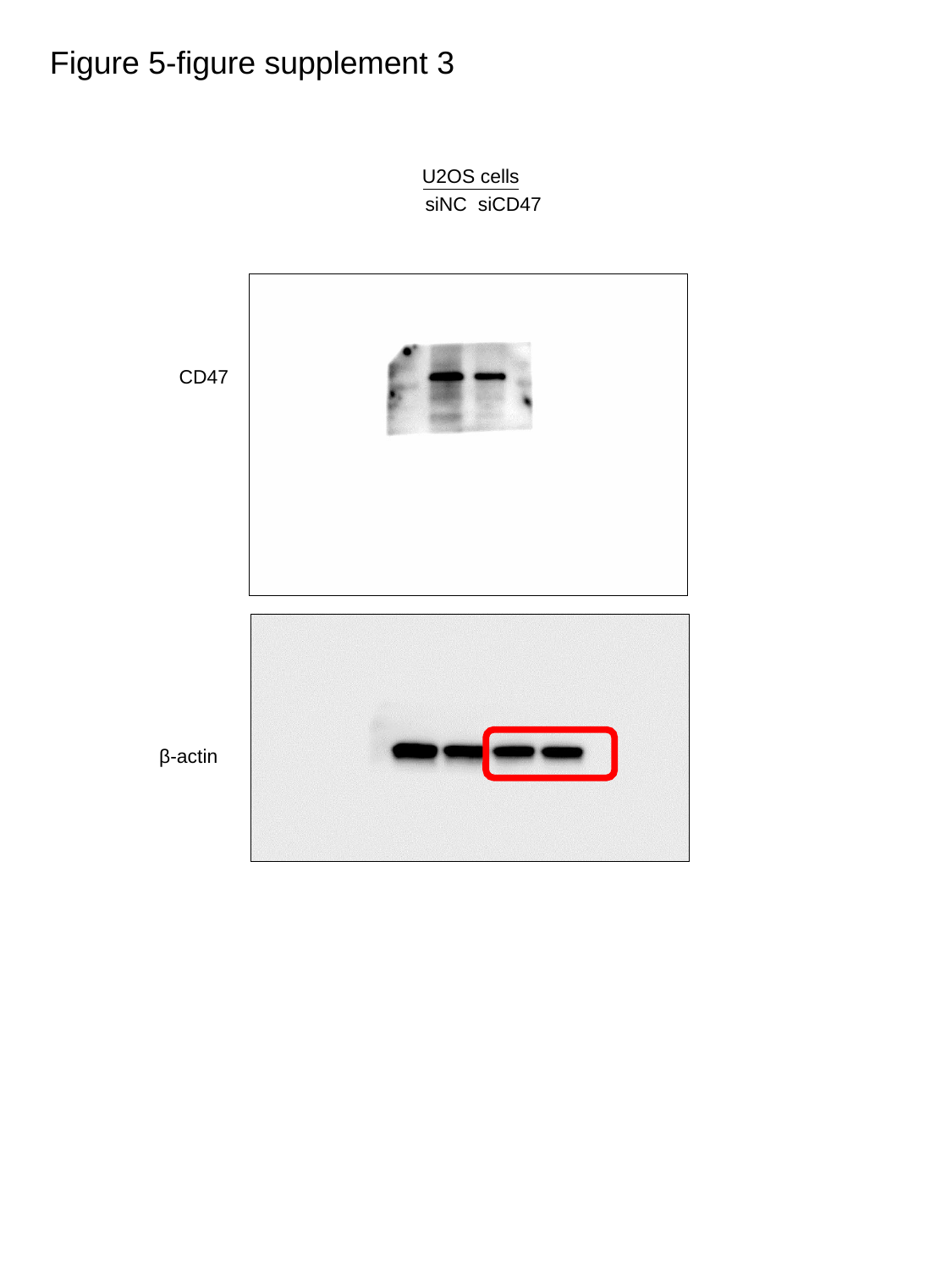

Figure 5-figure supplement 3
U2OS cells
siNC siCD47
CD47
β-actin

Supplement: Figure 5—figure supplement 3—source data 1. [file elife-83768-fig5-figsupp3-data1.zip › Figure 5-figure supplement 3-source data/Figure 5-figure supplement 3-source data 1/Figure 5-figure supplement 3-source data.pptx]

## Slide 1
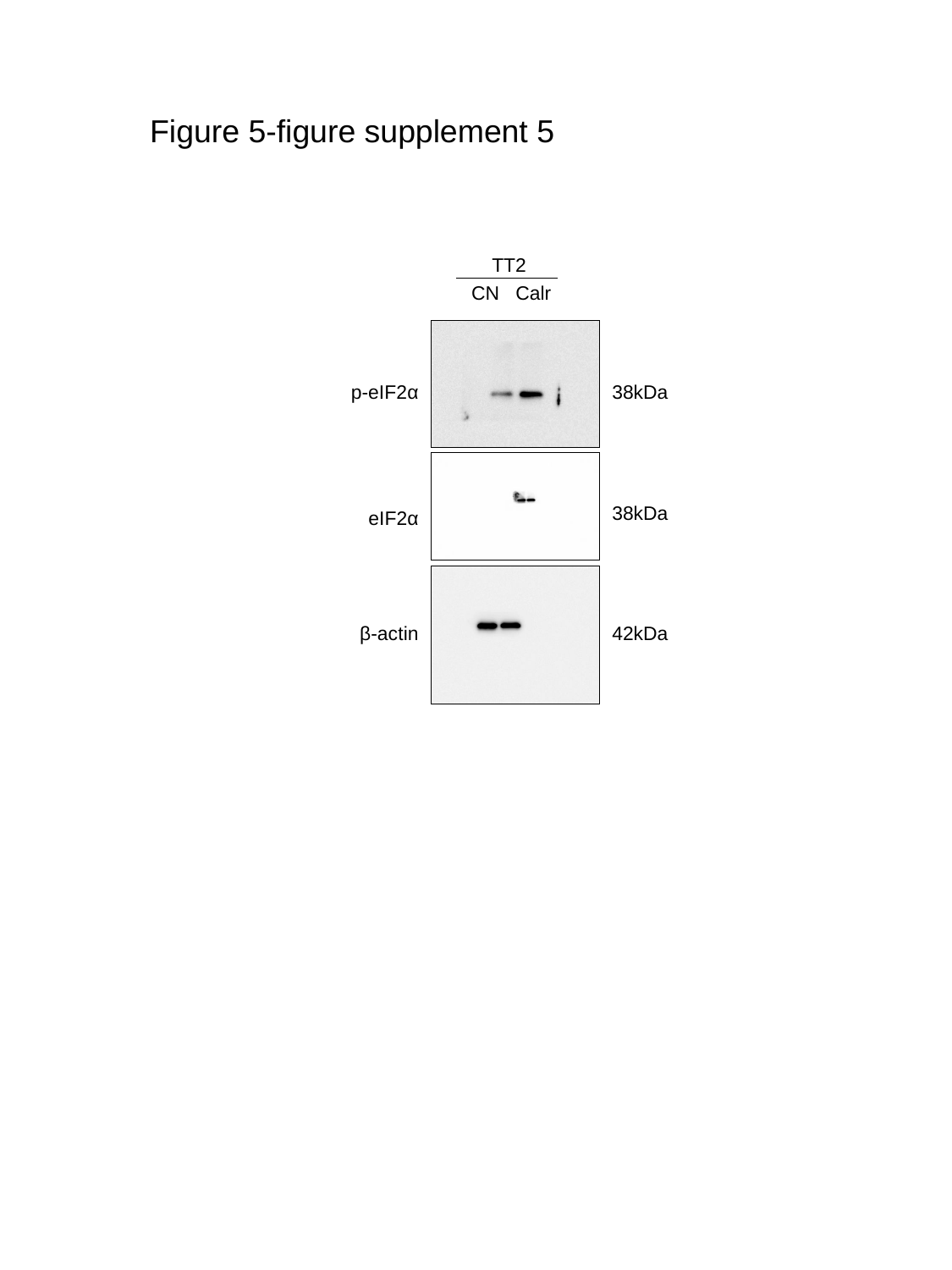

Figure 5-figure supplement 5
TT2
CN Calr
p-eIF2α
38kDa
38kDa
eIF2α
42kDa
β-actin

Supplement: Figure 5—figure supplement 5—source data 1. [file elife-83768-fig5-figsupp5-data1.zip › Figure 5-figure supplement 5-source data/Figure 5-figure supplement 5-source data 1/Figure 5-figure supplement 5-source data.pptx]

## Slide 1
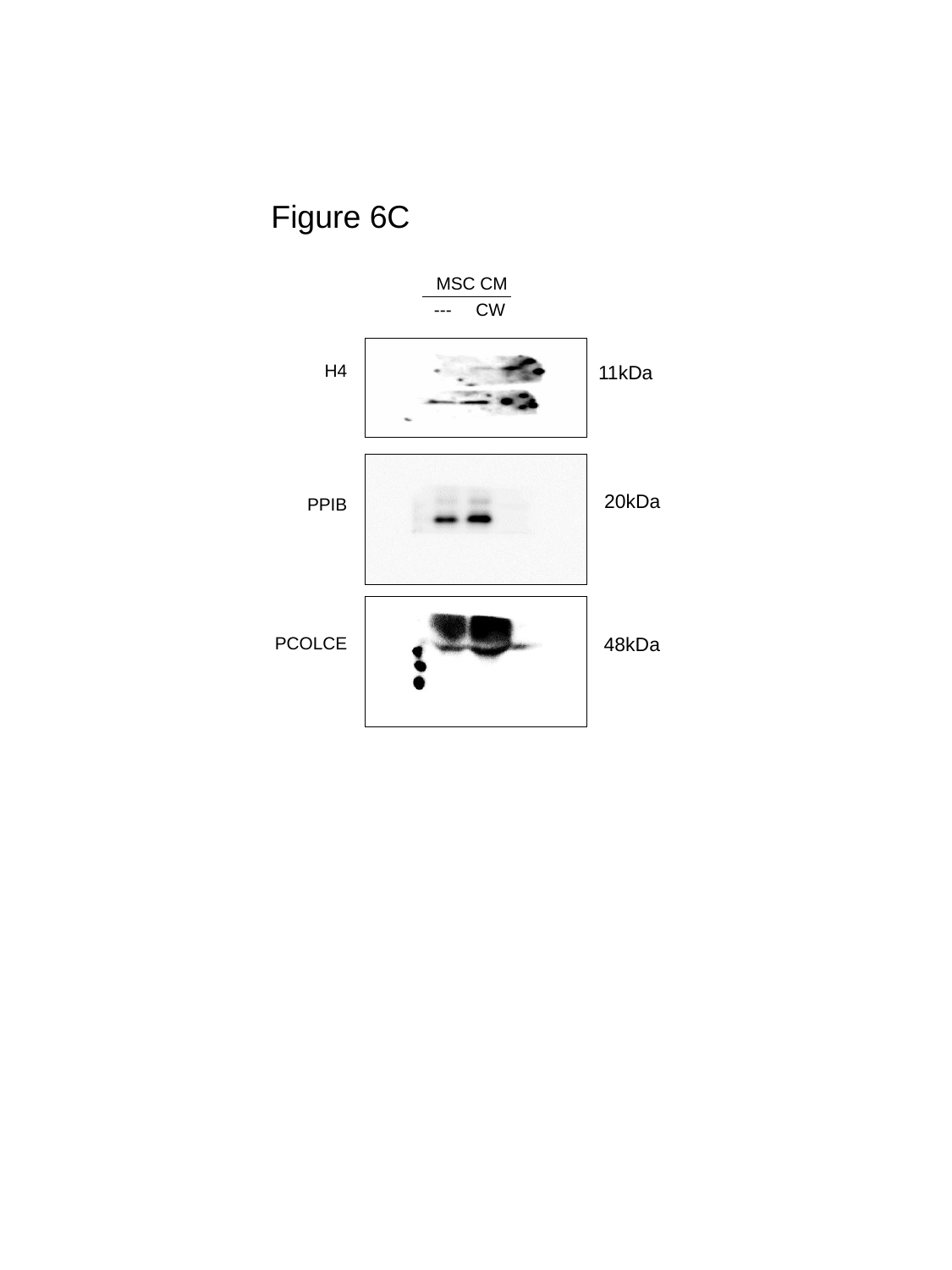

Figure 6C
MSC CM
--- CW
H4
11kDa
20kDa
PPIB
48kDa
PCOLCE

Supplement: Figure 6—source data 1. [file elife-83768-fig6-data1.zip › Figure 6 source data/Figure 6 source data1/Figure 6C-source data 4.pptx]

## Slide 1
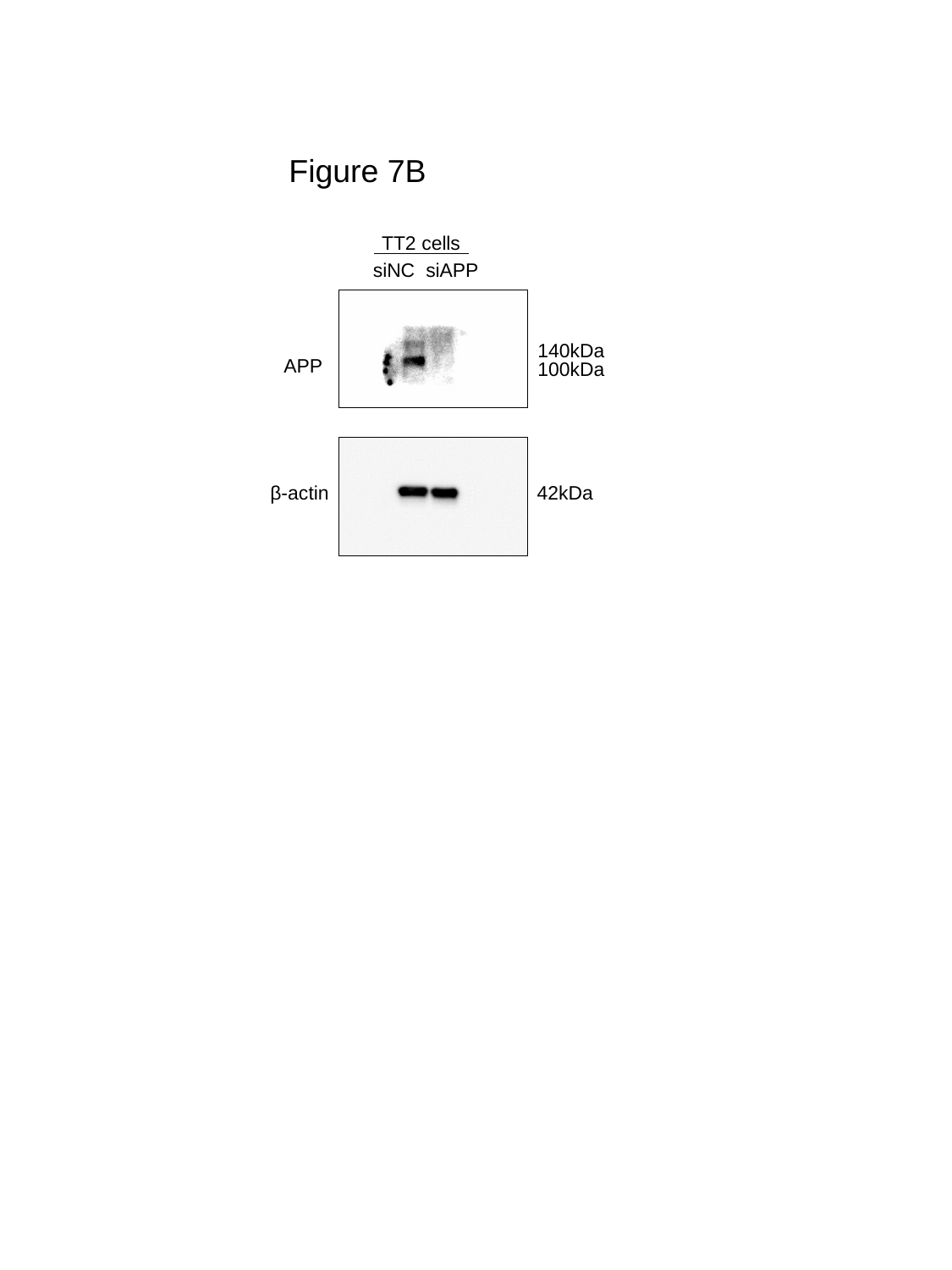

Figure 7B
TT2 cells
siNC siAPP
140kDa
APP
100kDa
β-actin
42kDa

Supplement: Figure 7—source data 1. [file elife-83768-fig7-data1.zip › Figure 7 source data/Figure 7B source data 1/Figure 7B-source data 3.pptx]

## Slide 1
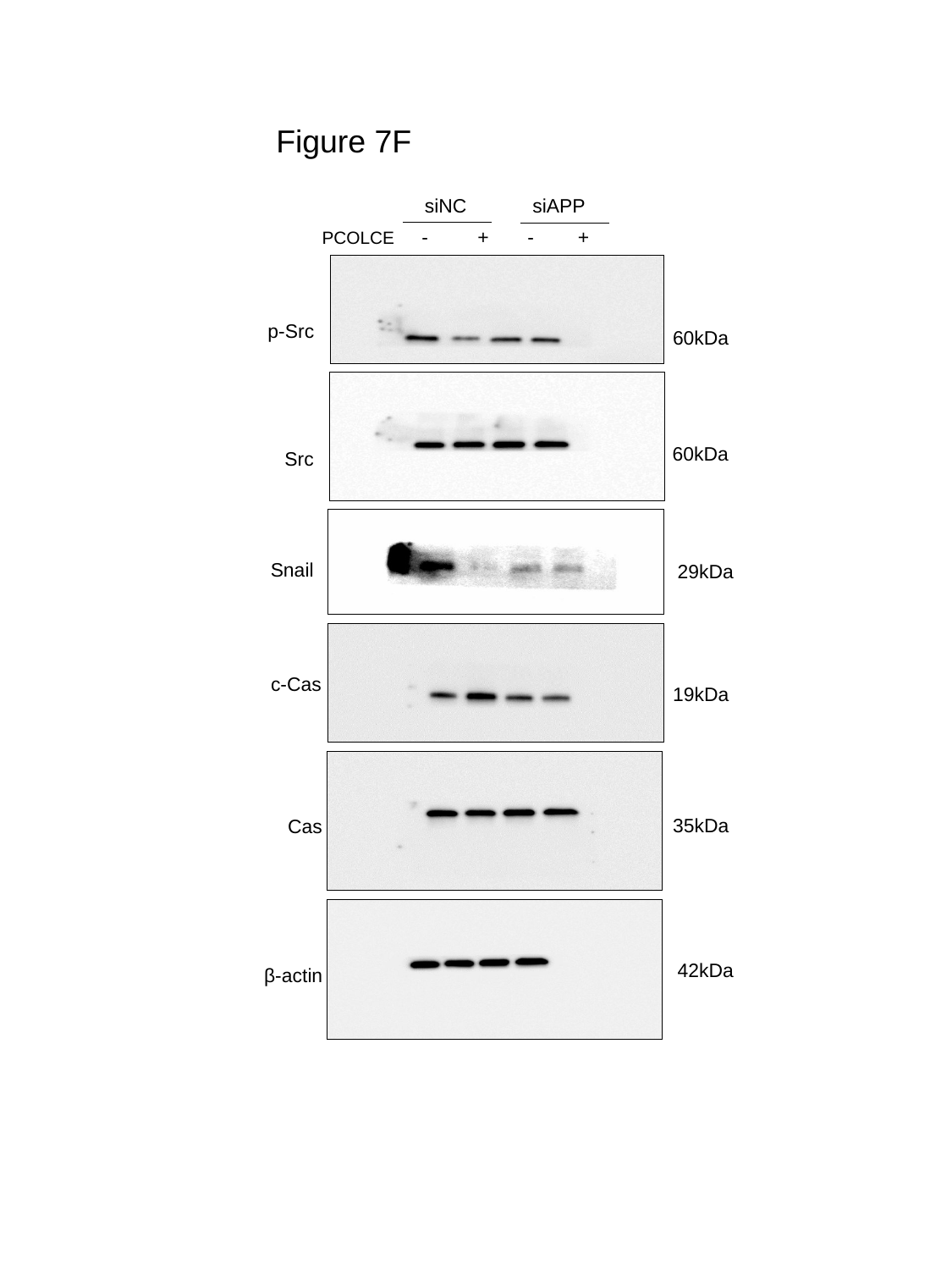

Figure 7F
siNC
siAPP
PCOLCE - + - +
p-Src
60kDa
60kDa
Src
Snail
29kDa
c-Cas
19kDa
35kDa
Cas
42kDa
β-actin

Supplement: Figure 7—source data 1. [file elife-83768-fig7-data1.zip › Figure 7 source data/Figure 7F souece data 1/Figure 7F-source data 7.pptx]
