# Supplementary material for: Osteosarcoma-enriched transcripts paradoxically generate osteosarcoma-suppressing extracellular proteins
Source: eLife. 2023 Mar 21;12:e83768. doi: 10.7554/eLife.83768 (PMC10030111; doi:10.7554/eLife.83768)
Supplement: Figure 5—source data 1. [file elife-83768-fig5-data1.zip › Figure 5-source data/Figure 5D-source data 1/Figure 5D-source data 5.pptx]

## Slide 1
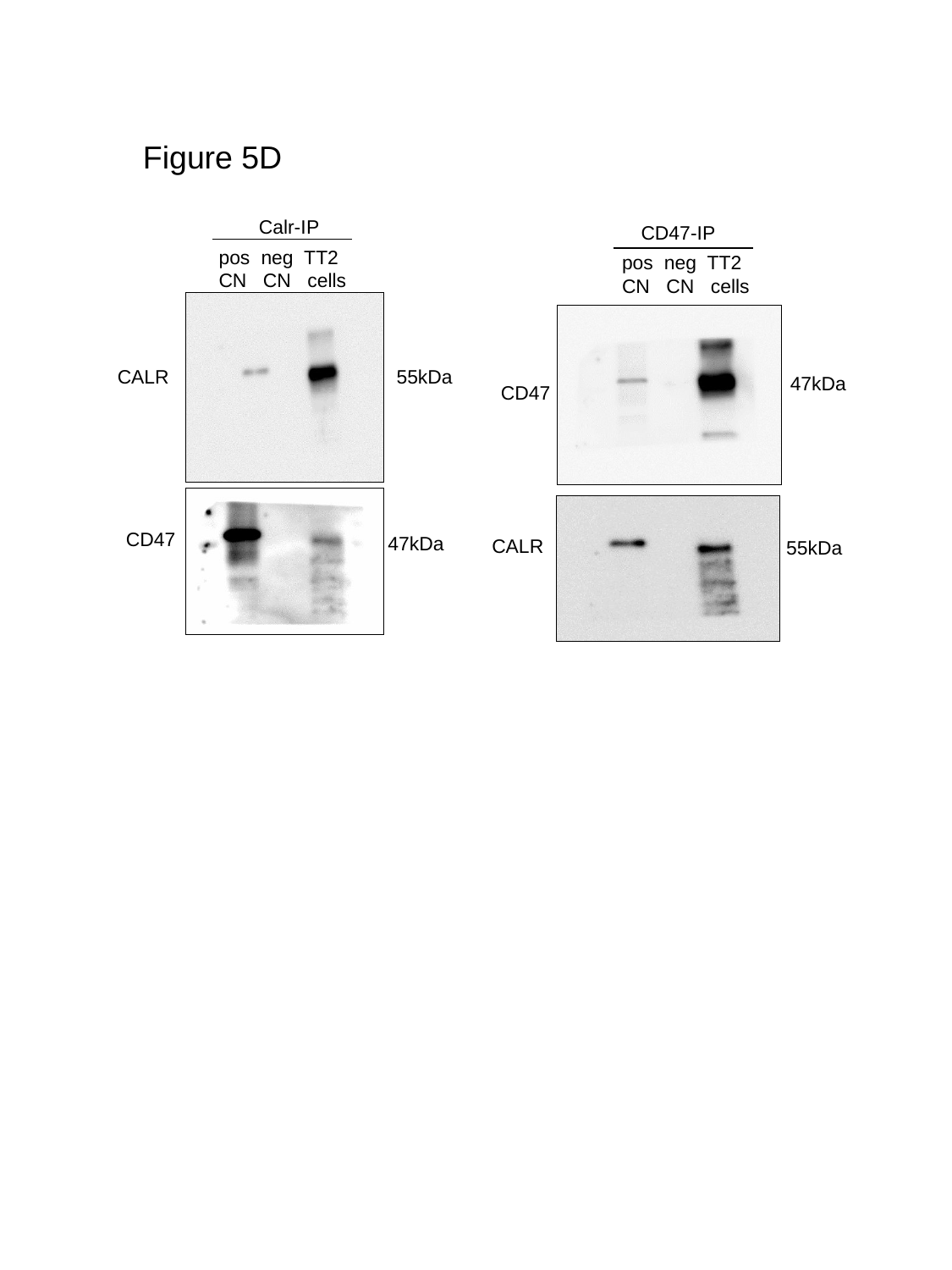

Figure 5D
Calr-IP
CD47-IP
pos neg TT2
CN CN cells
pos neg TT2
CN CN cells
55kDa
CALR
47kDa
CD47
CD47
47kDa
CALR
55kDa
