# Supplementary material for: Osteosarcoma-enriched transcripts paradoxically generate osteosarcoma-suppressing extracellular proteins
Source: eLife. 2023 Mar 21;12:e83768. doi: 10.7554/eLife.83768 (PMC10030111; doi:10.7554/eLife.83768)
Supplement: Figure 7—source data 1. [file elife-83768-fig7-data1.zip › Figure 7 source data/Figure 7A source data 1/Figure 7A-source data 5.pptx]

## Slide 1
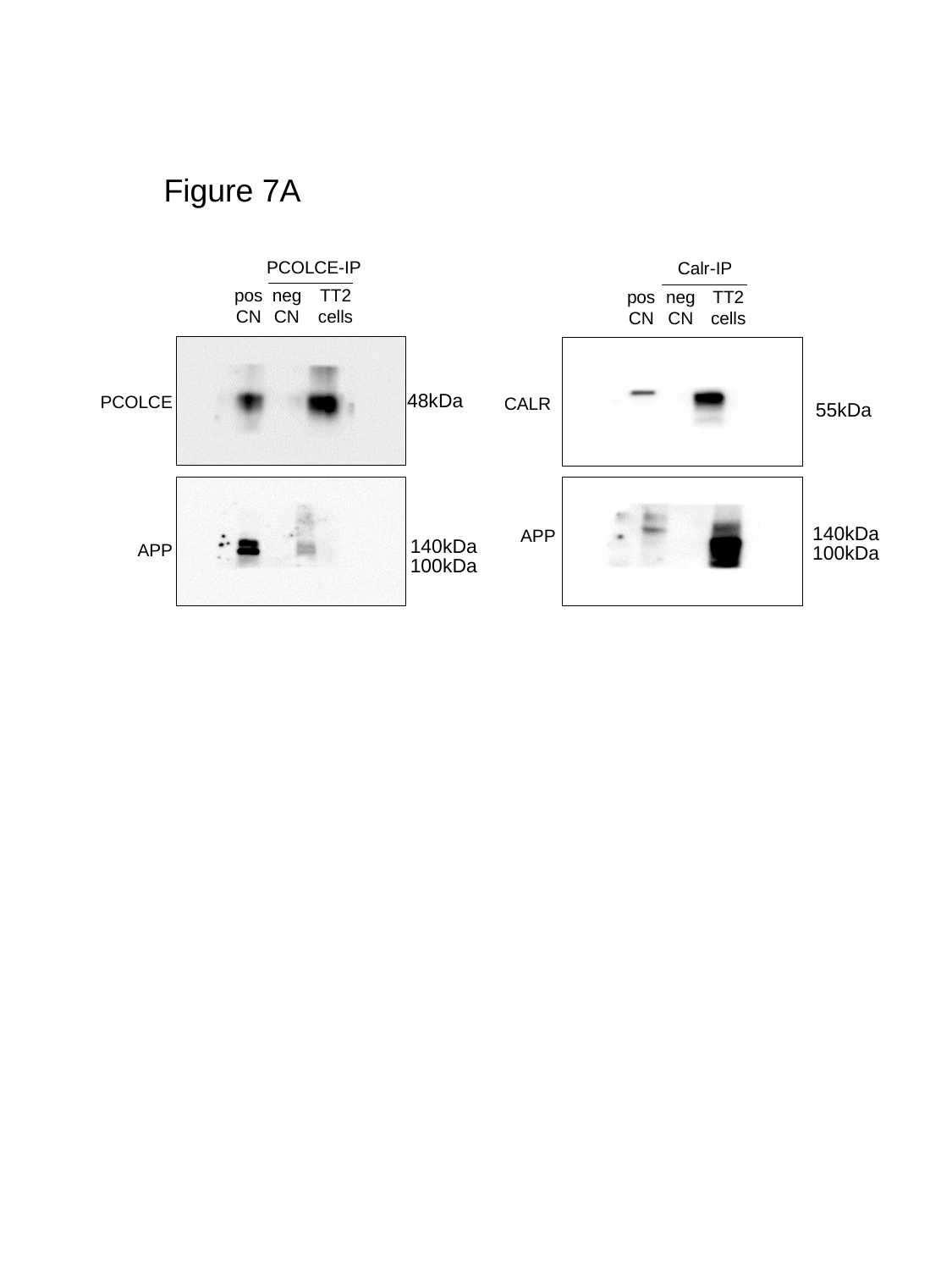

Figure 7A
PCOLCE-IP
Calr-IP
pos
CN
neg
CN
TT2
cells
pos
CN
neg
CN
TT2
cells
48kDa
PCOLCE
CALR
55kDa
140kDa
APP
140kDa
APP
100kDa
100kDa
