# Supplementary material for: Osteosarcoma-enriched transcripts paradoxically generate osteosarcoma-suppressing extracellular proteins
Source: eLife. 2023 Mar 21;12:e83768. doi: 10.7554/eLife.83768 (PMC10030111; doi:10.7554/eLife.83768)
Supplement: Supplementary file 1. [file elife-83768-supp1.docx]

Supplementary File 1: List of 7 selected proteins that were expressed higher in sarcoma tissues than the normal tissues in the TCGA dataset.

| Gene | Sarcoma  tissue | Control  tissue | Fold change |
| --- | --- | --- | --- |
| Enriched  in CM |  |  |  |
| CALR | 713.5 | 396.3 | 1.80 |
| ENO1 | 847.9 | 628.0 | 1.35 |
| HSP90ab1 | 456.8 | 311.5 | 1.47 |
| MSN | 238.4 | 102.7 | 2.32 |
| UBC | 1247.6 | 894.4 | 1.39 |
| Elevated  In OS |  |  |  |
| SPARC | 3330.4 | 795.3 | 4.19 |
| PCOLCE | 409.6 | 113.5 | 3.61 |
| CPE | 56.6 | 18.9 | 3.00 |
| GJA1 | 66.9 | 23.4 | 2.86 |
| S100A11 | 879.1 | 338.7 | 2.60 |
| H4 | 9.9 | 4.0 | 2.50 |
| PPIB | 784.1 | 379.1 | 2.07 |
